# Redox signaling in chronic airway diseases: pathogenic mechanisms and therapeutic implications

**DOI:** 10.3389/fphys.2026.1734890

**Published:** 2026-03-11

**Authors:** Mario Cazzola, Paola Rogliani, Luigino Calzetta, Frank M. P. van Haren, Clive Page, Maria Gabriella Matera

**Affiliations:** 1 Unit of Respiratory Medicine, Department of Experimental Medicine, University of Rome ‘Tor Vergata’, Rome, Italy; 2 Unit of Respiratory Clinical Pharmacology, Department of Clinical Science and Translational Medicine, University of Rome “Tor Vergata”, Rome, Italy; 3 Medical School, Australian National University, Canberra, ACT, Australia; 4 Intensive Care Unit, St George Hospital, Sydney, NSW, Australia; 5 Institute of Pharmaceutical Science, King’s College London, London, United Kingdom; 6 Unit of Pharmacology, Department of Experimental Medicine, University of Campania ‘Luigi Vanvitelli’, Naples, Italy

**Keywords:** Anti.oxidant agents, asthma, bronchiectasis, chronic obstructive pulmonary disease, long COVID, mucolytics, redox signal

## Abstract

Chronic airway diseases, including asthma, chronic obstructive pulmonary disease (COPD), and bronchiectasis, impose a significant global health burden. A central unifying feature of these diseases is redox imbalance, which is characterized by an excess of reactive oxygen and nitrogen species (ROS/RNS) that overwhelms the body’s antioxidant defenses, causing cellular dysfunction, inflammation, and tissue damage. Physiological ROS/RNS are essential for immune regulation and transcriptional control, but chronic oxidative stress disrupts these processes, driving disease progression. In asthma, eosinophil- and epithelial-derived ROS worsen airway hyperresponsiveness, induce mucus overproduction, and reduce steroid effects. COPD involves neutrophil-dominated inflammation, mitochondrial dysfunction, protease- and oxidant-mediated extracellular matrix degradation, and accelerated senescence. Bronchiectasis features persistent neutrophilic oxidative injury, microbial colonization, impaired mucociliary clearance, and progressive airway destruction. Exogenous oxidants, cigarette smoke, biomass fuels, pollutants, and pathogens further burden antioxidant systems, including superoxide dismutases, catalase, glutathione peroxidase, and Nrf2-regulated pathways. Redox dysregulation also contributes to post-COVID sequelae, promoting ongoing airway inflammation, fibrosis, and systemic complications. Therapeutic strategies targeting redox imbalance, mainly thiol-based antioxidants, Nrf2 activators, NADPH oxidase inhibitors, and mitochondria-targeted antioxidants, show mechanistic promise but face challenges in specificity, bioavailability, and clinical translation. Advancing precision redox medicine requires biomarker-guided patient stratification, high-resolution redox proteomics, single-cell and organoid models, and spatial imaging to identify disease-specific redox endotypes. Modulating pathological oxidative stress while preserving physiological signaling offers a novel avenue to improve outcomes. Understanding redox biology in airway disease highlights the potential of precision antioxidant strategies as adjuncts to conventional therapies, representing a paradigm shift in managing chronic airway disorders.

## Introduction

Chronic airway diseases, including asthma, chronic obstructive pulmonary disease (COPD), and bronchiectasis, constitute a major global health burden, with hundreds of millions affected and COPD ranking as the third leading cause of death worldwide (Zhai et al., 2025).

Asthma is a chronic inflammatory airway disease that is characterized by variable airflow obstruction and airway hyperresponsiveness (Global Initiative for Asthma, 2025). It affects approximately 260 million individuals worldwide (GBD, 2021 Asthma and Allergic Diseases Collaborators, 2025), with a global prevalence ranging from 1% to 29%, depending on age and geographic region (Yuan et al., 2025). The global incidence of asthma is estimated at 516.7 per 100,000 population, with higher rates observed in childhood and in high-income countries (GBD, 2021 Asthma and Allergic Diseases Collaborators, 2025). Asthma is defined by respiratory symptoms such as wheezing, shortness of breath, chest tightness, and cough. These symptoms and the degree of airflow limitation vary over time and are often triggered by exercise, exposure to allergens or irritants, weather changes, or viral respiratory infections. Airflow limitation may become persistent in some patients. Asthma is usually associated with chronic airway inflammation and hyperresponsiveness, even when symptoms are absent or lung function is normal. The etiology of asthma reflects a complex interaction between genetic predisposition and environmental exposures, including allergens, viral infections, air pollution, and tobacco smoke (Koppelman et al., 2025). The current standard of care is treatment with inhaled corticosteroids (ICS), often combined with long-acting β_2_-agonists (Cazzola et al., 2023; Global Initiative for Asthma, 2025). Biologic therapies targeting type 2 inflammation are reserved for patients whose symptoms are not controlled by standard therapy.

COPD is a progressive disorder characterized by persistent airflow limitation, chronic respiratory symptoms, and structural lung abnormalities, including emphysema and small airway disease (Global Initiative for Chronic Obstructive Lung Disease, 2026). It affects over 390 million people worldwide, primarily in low- and middle-income countries, with an estimated prevalence of 10%–13% among adults over 40 (Global Initiative for Chronic ObstructiveLung Disease, 2026; Adeloye et al., 2022). Although incidence rates are not routinely reported in global studies, longitudinal data from high-income countries suggest annual incidence rates of 0.5–1.0 cases per 100 person-years in adults over 40, with higher rates seen in smokers and those exposed to biomass fuels (Global Initiative for Chronic Obstructive Lung Disease, 2026; Adeloye et al., 2022). The primary etiological factor is long-term exposure to noxious particles, most commonly cigarette smoke, although biomass fuel exposure and air pollution are major contributors in low- and middle-income countries. Standard therapy includes inhaled bronchodilators (long-acting β_2_-agonists and muscarinic antagonists), ICSs in selected patients (primarily those with frequent exacerbations and elevated blood eosinophils), pulmonary rehabilitation, and long-term oxygen therapy in advanced disease (Global Initiative for Chronic ObstructiveLung Disease, 2026).

Bronchiectasis is characterized by irreversible bronchial dilatation, a chronic productive cough, recurrent respiratory infections, and neutrophilic airway inflammation (Barker and Karamooz, 2025). Its prevalence is increasing globally, particularly among older adults. Estimates range from 52 to over 1,000 cases per 100,000 population, depending on age, diagnostic criteria, and geographic region criteria (Nigro et al., 2024). Reported incidence rates of bronchiectasis range from 9.4 to 48.1 new cases per 100,000 person-years (Nigro et al., 2024). Etiology is heterogeneous and includes post-infectious damage, immunodeficiency, autoimmune disease, and impaired mucociliary clearance, although a substantial proportion of cases remain idiopathic despite extensive evaluation (O'Donnell, 2022; Barker and Karamooz, 2025). Diagnosis requires clinical symptoms and radiologic evidence of bronchial dilatation on high-resolution CT (O'Donnell, 2022). Bronchiectasis often coexists with severe asthma or COPD, leading to increased inflammation, exacerbations, and worse outcomes (Polverino et al., 2018). Management focuses on airway clearance techniques and pulmonary rehabilitation for patients with impaired exercise capacity (O’Donnell, 2022; Chalmers et al., 2025a). Long-term oral macrolide antibiotics are recommended for patients with ≥3 exacerbations per year, provided that nontuberculous mycobacterial infection has been ruled out. Inhaled antibiotics are considered for patients with chronic *Pseudomonas aeruginosa* infection and frequent exacerbations. Treatment of underlying causes is essential. Inhaled corticosteroids or bronchodilators (Martínez-García et al., 2022a; Martínez-García et al., 2022b) are reserved for patients with coexisting asthma or COPD.

A central unifying mechanism across asthma, COPD, and bronchiectasis is oxidative and nitrosative stress, reflecting an imbalance between reactive oxygen species (ROS) and reactive nitrogen species (RNS) generation and the capacity of antioxidant systems to neutralize them, leading to cellular dysfunction and tissue damage (Manful et al., 2025).

At physiological concentrations, these species serve as important signaling molecules. They regulate immune cell activation, transcription factor activity, and cell fate decisions through reversible oxidative post-translational modifications, particularly of cysteine residues on target proteins (Averill-Bates, 2024). This process, known as redox signaling, is essential for maintaining cellular homeostasis and coordinating adaptive responses to environmental changes (Kračun et al., 2025). However, when ROS and RNS are produced in excess and overwhelm antioxidant defenses, signaling specificity is lost. This imbalance leads to cellular dysfunction, tissue damage, and contributes to pathological processes such as chronic inflammation (Aramouni et al., 2023; Manful et al., 2025).

The dual role of these species is captured by the distinction between oxidative eustress (beneficial, low-level ROS/RNS signaling) and oxidative distress (harmful, chronic overproduction causing nonspecific biomolecular damage and disease progression) (Del Campo et al., 2023).

This narrative review highlights the role of redox signaling in asthma, COPD, and bronchiectasis and critically examines approaches aimed at improving the understanding of redox signaling in chronic airway diseases. Due to the introduction of novel redox perturbations by SARS-CoV-2 and post-SARS-CoV-2 infection syndromes, such as viral-mitochondrial interactions, endothelial oxidative injury, and pro-thrombotic biology, expanding redox-focused research and clinical discussion beyond classical chronic airway diseases is essential.

## Redox biology in the airways

Redox biology is a fundamental determinant of airway health and disease, as the lungs are continuously exposed to ROS and RNS from endogenous metabolism and environmental insults, counterbalanced by tightly regulated antioxidant defense systems (Table 1).

**TABLE 1:** Sources of oxidative/nitrosative stress and antioxidant defenses in the lung.

Category	Key players	Mechanism/role	References
ROS/RNS sources			
Endogenous	Mitochondrial, ETC (Complex I and III)	Leakage generates superoxide → converted to H_2_O_2_ by SOD2; regulates hypoxia and immunity	Araneda and Tuesta (2012) ; Elko et al. (2019)
	NADPH oxidases (NOX2, NOX4)	Controlled ROS production for host defense, signaling; NOX2 in phagocytes, NOX4 in airway/SMCs	van der Vliet et al. (2018) ; Michaeloudes et al. (2022)
	Xanthine oxidase	Produces superoxide and H_2_O_2_ during hypoxia-reoxygenation and inflammation	Araneda and Tuesta (2012)
	Nitric oxide synthases (eNOS, iNOS)	Generate NO; under inflammation, NO + O_2_• ^−^ → ONOO^−^, a potent oxidant	Araneda and Tuesta (2012) ; Di Paola and Cuzzocrea (2012)
	Heme	Catalyzes ROS formation *via* fenton chemistry; activates alveolar macrophages to produce NOX-dependent ROS and inflammation	Belcher et al. (2010); Simões et al. (2013)
Exogenous	Cigarette smoke	Contains ∼10^15^ oxidants/puff; gas + particulate radicals damage lipids, proteins, DNA; overwhelms antioxidants	Seo et al. (2023); Valavanidis et al. (2009) ; Cha et al (2023)
	Biomass fuel combustion	Releases ROS + particulate matter → airway inflammation, infections	Lakey et al. (2016)
	Industrial pollutants (heavy metals, VOCs)	Promote ROS directly or *via* cell interactions	Traboulsi et al. (2017)
	Ozone	Strong oxidant; induces lipid peroxidation, inflammatory signaling	Lee Y. G. et al. (2021); Valacchi et al. (2020)
	Pathogens (e.g., *P. aeruginosa*)	Phagocyte oxidative burst → ROS for killing; excessive ROS causes tissue damage, remodeling	Di Paola and Cuzzocrea (2012) ; Yoo et al. (2014)
Antioxidant defenses			
Enzymatic	Superoxide dismutases (SOD1, SOD2, SOD3)	Convert O_2_• ^−^ → H_2_O_2_, reduce ONOO^−^ formation, preserve NO	Jomova et al. (2024)
	Catalase	Converts H_2_O_2_ → H_2_O + O_2_ (peroxisomes)	Jomova et al. (2024)
	GPx	Detoxifies H_2_O_2_ and lipid peroxides using GSH	Jomova et al. (2024)
	Trx, peroxiredoxins	Reduce peroxides, maintain redox balance	Tebay et al. (2015)
	HO-1	Modulates ROS levels through antioxidant and pro-oxidant mechanisms; interacts with GSH metabolism and increases its levels	Ryter et al. (2007); Consoli et al. (2021)
Non-enzymatic	GSH	Major thiol antioxidant; direct radical scavenging + cofactor for detox enzymes	Cazzola et al. (2019)
	Uric acid	Plasma antioxidant, effective against ONOO^−^ and •OH	Whiteman et al. (2002)
	Vitamin C (ascorbate)	Water-soluble radical scavenger; regenerates vitamin E	Sies et al. (1992)
	Vitamin E (α-tocopherol)	Lipid-soluble scavenger of lipid peroxyl radicals; prevents lipid peroxidation	Niki (2014)
	Carotenoids (β-carotene, lycopene)	Quench singlet oxygen, scavenge peroxyl radicals in lipid domains	Black et al. (2020)
	Thiol-based drugs	Replenish GSH, direct antioxidant effects	Cazzola et al. (2019)
Regulatory pathways	Nrf2–Keap1 pathway	Oxidative stress modifies Keap1 cysteines → Nrf2 release → transcription of ARE-driven antioxidant genes	Baird and Yamamoto (2020); Lee J et al. (2021)

ARE, antioxidant response element; eNOS, endothelial nitric oxide synthase; ETC, electron transport chain; GPx, glutathione peroxidase; GSH, glutathione; HO-1, heme oxygenase-1; H_2_O_2_, hydrogen peroxide; iNOS, inducible nitric oxide synthase; Keap1, kelch-like ECH-associated protein 1; NADPH, nicotinamide adenine dinucleotide phosphate; NO, nitric oxide; Nrf2, nuclear factor-erythroid 2 p45-related factor 2; NOX, NADPH, oxidase; O_2_•^−^, superoxide anion; •OH, hydroxyl radical; ONOO^−^, peroxynitrite; RNS, reactive nitrogen species; ROS, reactive oxygen species; SMC, smooth muscle cell; SOD, superoxide dismutase; Trx, thioredoxin; VOC, volatile organic compound.

### Sources of ROS and RNS

The lung is highly dependent on redox regulation due to its continuous exposure to environmental oxidants and its intrinsic metabolic activity. Endogenous sources of ROS in the lung include mitochondrial electron transport chain leakage (primarily at complexes I and III), which generates superoxide that is converted to hydrogen peroxide (H_2_O_2_) by mitochondrial superoxide dismutase 2 (SOD2). These mitochondrial ROS contribute to the regulation of hypoxic responses and innate immunity (Araneda and Tuesta, 2012; Elko et al., 2019). Nicotinamide adenine dinucleotide phosphate (NADPH) oxidases (NOX family), especially NOX2 in neutrophils and macrophages and NOX4 in airway epithelial and smooth muscle cells, serve as regulated sources of ROS involved in host defense and cell signaling (van der Vliet et al., 2018; Michaeloudes et al., 2022). Xanthine oxidase is activated during hypoxia-reoxygenation and inflammation, producing superoxide and H_2_O_2_ (Araneda and Tuesta, 2012). Nitric oxide synthases (NOS), including endothelial (eNOS) and inducible (iNOS) isoforms, produce nitric oxide (NO), which can interact with superoxide to form reactive nitrogen species such as peroxynitrite (ONOO^−^) (Araneda and Tuesta, 2012; Di Paola and Cuzzocrea, 2012).

Exogenous ROS sources further increase the pulmonary redox burden. Cigarette smoke delivers an extraordinary number of oxidant molecules per puff, encompassing both gaseous and particulate radicals that interact with lipids, proteins, and nucleic acids (Valavanidis et al., 2009; Cha et al., 2023), overwhelming antioxidant defenses (Seo et al., 2023). Combustion of biomass fuels, including wood, crop residues, and coal, similarly releases ROS and particulate matter (PM) into the atmosphere (Lakey et al., 2016). Industrial pollutants, including heavy metals and volatile organic compounds (VOCs), promote ROS formation either directly or *via* interactions with cellular components (Traboulsi et al., 2017). Ozone, a common urban air pollutant, acts as a strong oxidant in the respiratory tract, inducing lipid peroxidation and activating inflammatory pathways (Lee Y. G. et al., 2021). Prolonged exposure to these environmental oxidants is consistently associated with increased oxidative burden, airway inflammation, and susceptibility to respiratory infections (Lakey et al., 2016; Valacchi et al., 2020).

Respiratory infection and colonization, exemplified by *P. aeruginosa* in bronchiectasis, stimulate robust production of ROS by host phagocytes, primarily neutrophils and macrophages, as part of the oxidative burst for pathogen clearance (Yoo et al., 2014). Although essential for host defense, excessive or dysregulated ROS production can contribute to tissue perturbation when dysregulated (Di Paola and Cuzzocrea, 2012).

An excess of ROS production and recurrent infections can also compromise the pulmonary microvasculature, resulting in red blood cell extravasation and the release of free heme into the lung parenchyma and airways (Ryter, 2021). Outside the protective environment of erythrocytes, heme acts as a potent pro-oxidant: its iron moiety catalyzes ROS formation *via* Fenton chemistry (Belcher et al., 2010) and, when incorporated into cell membranes, it activates alveolar macrophages to generate NOX-dependent ROS (Simões et al., 2013).

### Antioxidant defenses

Antioxidant defenses are critical in the lung because they counteract the continuous burden of ROS and RNS (Jomova et al., 2024). The main antioxidant defense systems in the airways comprise both enzymatic and non-enzymatic components, as well as regulatory pathways.

Enzymatic systems include superoxide dismutases (SODs), catalase, glutathione peroxidase (GPx), thioredoxin (Trx), and peroxiredoxins. SOD1 (cytosolic), SOD2 (mitochondrial), and SOD3 (extracellular) catalyze the dismutation of superoxide anion (O_2_•^−^) to H_2_O_2_, reducing the risk of ONOO^−^ formation and maintaining NO availability (Jomova et al., 2024). Catalase converts H_2_O_2_ to water and oxygen, primarily in peroxisomes. GPx reduces H_2_O_2_ and lipid peroxides using glutathione (GSH) as a substrate. Trx and peroxiredoxins reduce peroxides and maintain redox balance, often regulated by nuclear factor-erythroid 2 p45-related factor 2 (Nrf2) (Tebay et al., 2015).

Non-enzymatic systems include GSH, uric acid, vitamins C and E, and carotenoids. GSH is a major intracellular thiol antioxidant that directly neutralizes free radicals and serves as a cofactor for detoxification enzymes (Cazzola et al., 2019). Uric acid is a significant plasma antioxidant, particularly effective against ONOO^−^and hydroxyl radicals (Whiteman et al., 2002). Vitamin C (ascorbate) is a water-soluble antioxidant that scavenges free radicals in aqueous compartments and directly or indirectly reduces the tocopheroxyl radical, thereby regenerating vitamin E (Sies et al., 1992). Vitamin E is the most abundant and important lipophilic radical-scavenging antioxidant *in vivo*, acting as a primary scavenger of lipid peroxyl radicals and inhibiting lipid peroxidation in biological membranes (Niki, 2014). Carotenoids (e.g., β-carotene, lycopene) are efficient quenchers of singlet oxygen and can scavenge peroxyl radicals, with these antioxidant activities occurring within the lipid domains where carotenoids reside (Black et al., 2020).

The Nrf2-Kelch-like ECH-associated protein 1 (Keap1) pathway is the principal regulatory system for antioxidant gene transcription (Baird and Yamamoto, 2020). Under homeostatic conditions, Keap1 binds Nrf2 in the cytoplasm and targets it for ubiquitin-mediated proteasomal degradation, thereby maintaining low basal Nrf2 activity. Upon exposure to oxidative or electrophilic stress, critical cysteine residues on Keap1 are modified, which disrupts its ability to target Nrf2 for degradation (Lee J. et al., 2021). This allows Nrf2 to accumulate, translocate to the nucleus, and activate transcription of antioxidant response element (ARE)-driven genes, including those encoding for heme oxygenase-1 (HO-1), nicotinamide adenine dinucleotide phosphate (reduced form) quinone oxidoreductase 1, and glutamate-cysteine ligase catalytic subunit, among others. In chronic airway diseases such as COPD, asthma, and idiopathic pulmonary fibrosis, Nrf2 activity is frequently impaired (Lee J. et al., 2021).

HO-1 also plays a role in the antioxidant defense system of the lung. HO-1 is an inducible enzyme that is upregulated in response to ROS and other oxidative stressors, such as infection, hyperoxia, and exposure to pollutants (Ma et al., 2025). Its activity modulates ROS levels through antioxidant and pro-oxidant mechanisms (Ryter et al., 2007). HO-1 degrades pro-oxidant heme into biliverdin, carbon monoxide (CO), and ferrous iron (Fredenburgh et al., 2007). This reaction eliminates free heme and generates metabolites with significant cytoprotective properties. Biliverdin and its reduction product, bilirubin, function as powerful free radical scavengers, while CO exerts anti-inflammatory, anti-apoptotic, and vasodilatory effects that help preserve pulmonary integrity (Ryter et al., 2007). Concurrently, the iron released from heme breakdown stimulates ferritin synthesis, sequestering labile iron and preventing iron-driven oxidative injury (Balla et al., 2005). HO-1 also interacts with GSH metabolism, as its products and regulatory pathways can increase GSH levels, further strengthening the antioxidant capacity of the lung (Consoli et al., 2021).

## Pathogenic consequences of redox imbalance

**FIGURE 1:**
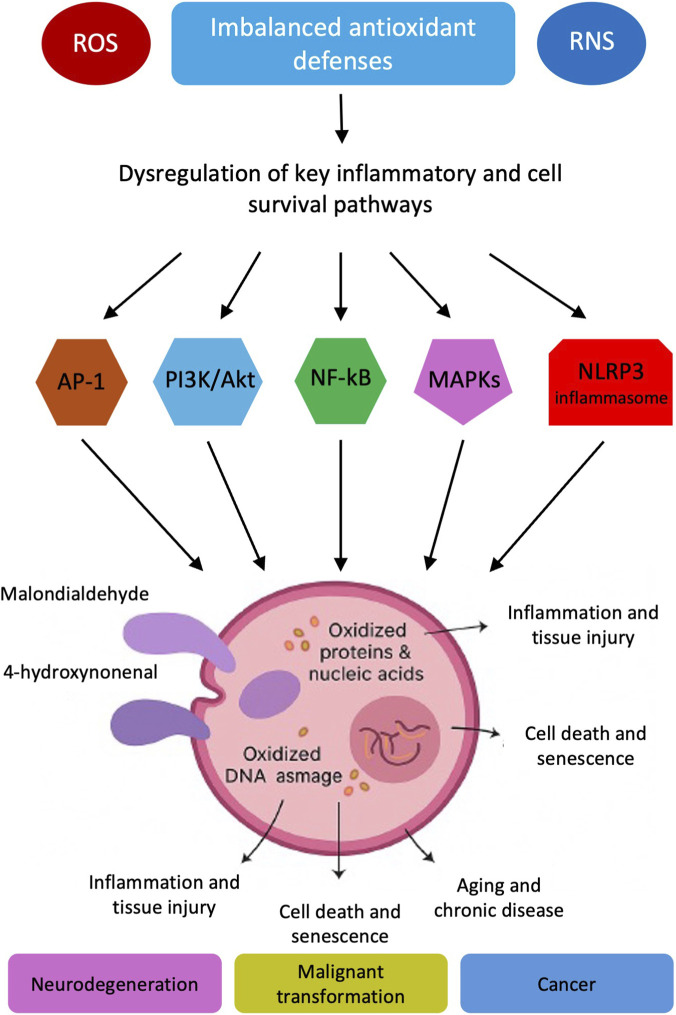
Redox imbalance and its pathogenic consequences. Excess reactive oxygen and nitrogen species (ROS/RNS) resulting from impaired antioxidant defenses activate key inflammatory and cell survival pathways (NF-κB, AP-1, MAPKs, PI3K/Akt, and NLRP3 inflammasome). These cascades promote oxidative damage to lipids, proteins, and DNA, leading to inflammation, senescence, and cell death, ultimately contributing to aging, neurodegenerative diseases, cancer, and other chronic conditions.

Excessive ROS and/or RNS, resulting from an imbalance in antioxidant defenses, have been shown to trigger pathogenic consequences by dysregulating key inflammatory and cell survival pathways (Figure 1).

Redox imbalance drives pathological inflammation, immune dysregulation, and cell death by modulating nuclear factor kappa-light-chain-enhancer of activated B cells (NF-κB), activator protein-1 (AP-1), mitogen-activated protein kinases, phosphoinositide 3-kinase (PI3K)/Akt, and NOD-like receptor protein 3 (NLRP3) inflammasome activation, with complex crosstalk and feedback mechanisms amplifying disease processes (Rius-Pérez et al., 2020; Morris et al., 2022).

Other consequences of redox imbalance include a spectrum of molecular and cellular dysfunctions that underlie the pathophysiology of numerous chronic diseases, by promoting cell death, senescence, and dysregulated inflammation. Lipid peroxidation creates aldehydes like 4-hydroxynonenal and malondialdehyde (MDA) that harm cell membranes, disrupt ions, and change how proteins work (Barrera et al., 2018). These aldehydes trigger inflammation and tissue injury. They also change proteins and nucleic acids, affecting how cells work. This can lead to aging, neurodegenerative diseases, and cancer. Oxidized bases, strand breaks, and cross-links from deoxyribonucleic acid (DNA) oxidation can lead to cellular senescence, impaired DNA repair, and malignant transformation (Hoeijmakers, 2009). Oxidative DNA damage drives mutagenesis and genomic instability. Protein nitration and oxidation can inactivate enzymes and alter signaling pathways, impacting cell survival, inflammation, and neurodegeneration (Moldogazieva et al., 2018).

## Redox signaling in asthma

Asthma is a heterogeneous disease with phenotypes ranging from allergic type (T)2-high asthma to non-eosinophilic T2-low disease (Woodruff et al., 2009). Oxidative stress contributes to airway hyperresponsiveness, mucus hypersecretion, airway remodeling, and treatment resistance (Michaeloudes et al., 2022).

### Mechanistic insights

Eosinophils contribute to airway inflammation in asthma, in part through ROS production via eosinophil peroxidase and NADPH oxidase (Andreadis et al., 2003). Oxidative stress increases the expression of epithelial-derived alarmins and chemokines, which further attract and activate Th2 cells and innate lymphoid cells, maintaining the T2 inflammatory environment (O'Grady and Kita, 2023). The toxic effects of eosinophil peroxidase-generated oxidants and NADPH oxidase-derived superoxide damage epithelial cells, increase barrier permeability, and stimulate mucus overproduction, all of which are key features of asthma pathology (Kleniewska and Pawliczak, 2017). This epithelial damage also facilitates allergen penetration and sustains chronic airway inflammation (Steffan et al., 2024).

The upregulation of iNOS increases NO production (Pacher et al., 2007). NO then combines with superoxide to generate ONOO^−^, which drives nitrosative stress and cellular injury.

ROS, specifically H_2_O_2_, induce airway smooth muscle contraction by increasing calcium influx and activating the Rho-kinase pathway, thereby contributing to airway hyperresponsiveness (Kojima et al., 2007).

Oxidative stress suppresses histone deacetylase 2 (HDAC2) activity primarily through post-translational modifications, including nitration and carbonylation of critical amino acid residues, resulting in diminished HDAC2 expression and enzymatic function (Osoata et al., 2009b). The consequent loss of HDAC2 compromises the ability of corticosteroids to recruit this enzyme to inflammatory gene promoters, thereby impairing histone deacetylation and the repression of pro-inflammatory transcription, ultimately driving corticosteroid resistance (Osoata et al., 2009b).

In neutrophilic asthma, these effects are compounded by an imbalance in chromatin-modifying enzymes, characterized by increased histone acetyltransferase (HAT) activity and reduced HDAC activity (Gunawardhana et al., 2014). This elevated HAT/HDAC ratio establishes a transcriptional milieu that sustains chronic inflammation and confers poor corticosteroid responsiveness (Osoata et al., 2009b). Smoking-related asthma and passive smoke exposure further exacerbate this process by reducing HDAC2 through activation of the PI3K/Akt pathway, thereby aggravating corticosteroid insensitivity (Osoata et al., 2009b; Kobayashi et al., 2014a). In severe eosinophilic asthma, impairment of HDAC2 is generally less pronounced; however, PI3K pathway activation and diminished HDAC activity may still contribute to relative steroid insensitivity in a subset of patients (Kobayashi et al., 2014b). Nevertheless, in classic eosinophilic asthma, HDAC2 levels and activity are usually preserved, and corticosteroid sensitivity remains intact (Osoata et al., 2009b).

### Biomarkers of oxidative stress in asthma

The biomarkers of oxidative stress in asthma include several well-characterized molecules measured in various biological samples. Collectively, these biomarkers provide insight into the oxidative and nitrosative stress burden, aiding in disease phenotyping and monitoring.

Elevated fractional exhaled nitric oxide (FeNO) reflects increased airway inflammation and oxidative/nitrosative stress and is widely used in clinical practice for monitoring asthma patients (Chamitava et al., 2020). FeNO is a surrogate for iNOS activity in airway epithelial cells. 8-isoprostane is a stable product of lipid peroxidation and is consistently increased in the exhaled breath condensate of asthma patients, correlating with disease severity and poor control (Aldakheel et al., 2016). It is a reliable marker of oxidative damage in the airways. MDA is a byproduct of polyunsaturated fatty acid peroxidation and is elevated in both plasma and exhaled breath condensate of asthmatic individuals, especially in those with uncontrolled or severe disease (Corradi et al., 2003; Karadogan et al., 2022). Higher MDA levels are associated with increased oxidative stress and worse asthma control. GSH is a key antioxidant, and a lower GSH/oxidized GSH (GSSG) ratio indicates oxidative stress (Averill-Bates, 2024). Asthma patients, particularly those with poor control, show decreased GSH and increased GSSG in the epithelial lining fluid, reflecting impaired antioxidant defenses (Fitzpatrick et al., 2009). ONOO^−^is formed from the reaction of nitric oxide and superoxide, leading to nitration of tyrosine residues (e.g., nitrotyrosine) in proteins (Reiter et al., 2000). These modified proteins are elevated in sputum from asthmatic patients and serve as markers of nitrosative stress (Hanazawa et al., 2000).

## Redox signaling in COPD

COPD is strongly linked to oxidative stress from cigarette smoke, environmental exposures, and endogenous ROS production (Barnes, 2020).

### Mechanistic insights

Cigarette smoke is a major exogenous source of oxidants driving the pathogenesis of COPD (Valavanidis et al., 2009; Cha et al., 2023; Seo et al., 2023). These oxidants trigger oxidative and carbonyl stress, which contribute to airway inflammation, epithelial injury, and mitochondrial dysfunction, all of which are central to COPD pathogenesis (Yao and Rahman, 2011).

In COPD, persistent inflammation of the airways and lung parenchyma is marked by the recruitment and activation of neutrophils, macrophages, and lymphocytes (Barnes, 2020). These immune cells represent key sources of ROS, which play a central role in mediating tissue injury and accelerating disease progression. The combined action of cell-derived oxidants, including superoxide anion, H_2_O_2_, and hypochlorous acid, creates a sustained oxidative burden that promotes airway remodeling, drives emphysematous changes, and contributes to the development of corticosteroid resistance in COPD (Yao and Rahman, 2011).

Mitochondrial dysfunction, specifically reduced mitophagy leading to the accumulation of dysfunctional, ROS-generating mitochondria, plays a central role in the pathogenesis of COPD (Antunes et al., 2021). The accumulation of these damaged mitochondria amplifies oxidative stress, promotes cellular senescence, and triggers pro-inflammatory signaling cascades. This process contributes to epithelial cell death, tissue remodeling, and emphysematous changes characteristic of COPD (Zhou et al., 2021).

In COPD, oxidative and nitrative stress induces extensive post-translational modifications of HDAC2, including tyrosine nitration and subsequent ubiquitin-proteasome-mediated degradation (Barnes, 2009). These processes lead to a profound and sustained reduction in HDAC2 activity and expression within alveolar macrophages and lung tissue, representing a central mechanism underlying the pronounced corticosteroid resistance characteristic of COPD (Barnes, 2009). The loss of HDAC2 is both persistent and widespread, closely correlating with disease severity. Thus, COPD is characterized by a more uniform and severe reduction in HDAC2 due to chronic oxidative/nitrative stress, while in asthma, HDAC2 impairment is phenotype-dependent and most notable in severe, neutrophilic, or smoking-related cases.

ROS activate matrix metalloproteinases (MMPs) and contribute to matrix destruction and the development of emphysema in COPD by directly modifying the structure of MMPs and their endogenous inhibitors, as well as by upregulating MMP gene expression (Christopoulou et al., 2023). In addition, ROS inactivate tissue inhibitors of metalloproteinases (TIMPs), further tipping the protease-antiprotease balance toward unchecked proteolysis and matrix breakdown (Wang et al., 2007).

ROS-induced telomere shortening and cellular senescence are central mechanisms of accelerated aging in patients with COPD. Chronic exposure to exogenous and endogenous ROS leads to oxidative damage of telomeric DNA, which is highly sensitive to such injury (Opresko et al., 2025). This accelerates telomere attrition and telomere dysfunction, triggering a DNA damage response at chromosome ends and activating cell cycle arrest pathways (such as p53/p21, p16) that result in cellular senescence.

### Biomarkers of oxidative stress in COPD

Biomarkers that reflect the redox imbalance provide critical insights into disease activity, phenotyping, and therapeutic response. They correlate with lung function decline (FEV_1_ reduction) and radiographic emphysema, highlighting their role as potential indicators of disease progression (Fratta et al., 2020). Furthermore, increased oxidative stress markers are consistently observed during acute exacerbations, supporting their role in exacerbation prediction and monitoring (Zinellu et al., 2021).

There are systemic and airway biomarkers. Elevated levels of H_2_O_2_ in exhaled breath condensate are a validated biomarker of oxidative stress in COPD (Dekhuijzen et al., 1996). Increased concentrations of 8-hydroxy-2′-deoxyguanosine (8-OHdG) in both urine and serum are established markers of oxidative DNA damage in patients with COPD (Cao et al., 2022). Higher urinary levels of F2-isoprostanes (specifically iPF2α-III) are a robust index of oxidant stress in COPD (Praticò et al., 1998), whilst lower plasma/serum vitamin C (Calikoğlu et al., 2002) and reduced GSH (Sotgia et al., 2020) levels are consistently observed in COPD patients.

Plasma vitamin C and reduced GSH are available clinically at major reference labs; urinary F_2_-isoprostanes and urinary 8-OHdG are available from reference/specialty labs (often by liquid chromatography–tandem mass spectrometry) but are not routine in every hospital. H_2_O_2_ in exhaled breath condensate remains largely a research marker because of lack of standardized collection/analysis and limited clinical reference range (Stolarek et al., 2010).

## Redox signaling in bronchiectasis

Bronchiectasis is characterized by irreversible airway dilatation, chronic infection, and neutrophil-dominated inflammation, with redox imbalance central to the vicious cycle of damage (Qin et al., 2021).

### Mechanistic insights

The pathogenesis of bronchiectasis is driven by a complex interplay of neutrophilic inflammation, protease-oxidant interactions, persistent microbial colonization, and impaired mucociliary clearance. Neutrophil-derived ROS, primarily generated *via* NADPH oxidase and myeloperoxidase, induce airway tissue injury and sustain inflammatory signaling. In bronchiectatic airways, neutrophils exhibit delayed apoptosis, increased myeloperoxidase release, and impaired bactericidal capacity, collectively perpetuating inflammation and promoting extracellular matrix degradation through elevated neutrophil elastase activity (Chalmers et al., 2025b).

Persistent pathogens, notably *P. aeruginosa*, further amplify oxidative stress by stimulating epithelial ROS production, facilitating biofilm formation and bacterial persistence, which impedes clearance and maintains chronic infection (Chalmers and Hill, 2013). The protease-oxidant axis represents a central mechanism, as ROS potentiate neutrophil elastase activity, inducing a protease-antiprotease imbalance that drives progressive airway destruction (Chalmers et al., 2025a).

Impaired mucociliary clearance compounds this pathogenic cycle. Oxidative injury to the ciliated epithelium reduces ciliary beat frequency, sustaining mucus stasis, a hallmark of bronchiectasis (O'Donnell A.E., 2022). Mucus retention fosters local hypoxia and increases mucus viscoelasticity, exacerbating airway obstruction and predisposing to recurrent infection (Barker and Karamooz, 2025). Concurrently, inflammasome activation emerges as a pivotal contributor to disease progression. ROS prime the NLRP3 inflammasome, enhancing IL-1β production, which correlates with disease severity and modulates mucin expression, thereby promoting mucus thickening and airway plugging (Barker and Karamooz, 2025; Doumat et al., 2025).

### Biomarkers of oxidative stress in bronchiectasis

Several biomarkers have been studied to measure oxidative stress in bronchiectasis (Johnson et al., 2024). Lipid peroxidation products such as MDA, 4-hydroxynonenal (4-HNE), and F2-isoprostanes are usually measured in sputum, plasma, and exhaled breath condensate (Tsikas, 2017). MDA and F2-isoprostanes are well-established markers in both sputum and exhaled breath condensate, with higher levels often found in sputum compared to exhaled breath condensate, reflecting local airway oxidative stress (Corradi et al., 2004). 4-HNE can be measured in both sputum and exhaled breath condensate, although its quantification is technically difficult due to rapid metabolism (Horváth et al., 2005). Protein oxidation markers like advanced oxidation protein products (AOPP), protein carbonyls, and nitrotyrosine are most commonly measured in plasma and sputum, indicating oxidative modifications of proteins (Kehm et al., 2021). Protein carbonyls and AOPP are established plasma markers (Gryszczyńska et al., 2017), while nitrotyrosine is detected in sputum and bronchoalveolar lavage fluid as a marker of nitrosative stress (Antus, 2016). Sputum 8-OHdG reflects local airway DNA oxidation, whereas measurements in plasma/serum and urine provide systemic indicators of oxidative DNA damage (Tzortzaki et al., 2012).

The antioxidant defense system can be assessed through enzymatic markers such as SOD, GPx, and catalase, as well as non-enzymatic components like GSH and vitamins C and E (Demirci-Çekiç et al., 2022). Catalase activity is often reduced, and SOD activity is decreased in the plasma of bronchiectasis patients compared to healthy controls, indicating impaired enzymatic antioxidant defenses (de Camargo et al., 2021). The depletion of GSH in monocytes and airway samples is associated with increased oxidative stress in bronchiectasis (de Camargo et al., 2021). Vitamin C and E levels, though less frequently measured in bronchiectasis, are established systemic antioxidants and their reduction is a marker of chronic oxidative stress in chronic airway diseases (Demirci-Çekiç et al., 2022).

Clinically, higher oxidative stress biomarker levels are consistently associated with worse clinical severity, evidence of greater pathological changes in the lung assessed radiologically, and increased exacerbation frequency in bronchiectasis (Chalmers et al., 2017).

## Redox signaling and oxidative stress across asthma, COPD, and bronchiectasis

Although redox signaling and oxidative stress are shared mechanisms across asthma, COPD and bronchiectasis, they also exhibit disease-specific differences (Table 2). Shared redox signaling pathways in asthma, COPD, and bronchiectasis converge on the inflammation–oxidation axis, wherein ROS activate redox-sensitive transcription factors, notably NF-κB and AP-1, thereby driving the transcription of pro-inflammatory genes and sustaining chronic airway inflammation (Rius-Pérez et al., 2020; Zuo and Wijegunawardana, 2021; Morris et al., 2022). This pathogenic cycle is initiated by both exogenous oxidants and endogenous ROS generated from activated neutrophils, eosinophils, and macrophages, ultimately leading to persistent oxidative stress in these airway diseases (Albano et al., 2022).

**TABLE 2:** Redox signaling and oxidative stress across asthma, COPD, and bronchiectasis. Shared mechanisms and disease-specific differences.

Mechanism/Pathway	Key players	Effects on airway disease	Disease-specific features	Clinical implications
Redox signaling and transcription	ROS, NF-κB, AP-1	Activation of pro-inflammatory genes; sustains chronic airway inflammation	Asthma: Type 2 inflammation (eosinophil-driven) COPD: Neutrophil-driven inflammation; emphysema	Antioxidant therapy may reduce chronic inflammation
Sources of ROS	Exogenous: Pollutants, cigarette smoke Endogenous: Neutrophils, eosinophils, macrophages	Initiates oxidative stress cycle	Asthma: Mostly eosinophils COPD: Cigarette smoke major contributor Bronchiectasis: Chronic infection-driven neutrophil activation	Risk factor modification; infection control
Mucus hypersecretion	ROS, MUC5AC, MUC5B, NF-κB, AP-1	Airway obstruction; impaired mucociliary clearance	Asthma: Episodic mucus plugging COPD: Chronic bronchitis phenotype Bronchiectasis: Copious, persistent purulent sputum	Symptom control; mucolytics, airway clearance strategies
Barrier dysfunction	ROS, tight junction proteins (occludin, claudins)	Increased epithelial permeability; higher infection risk; amplifies inflammation	Asthma: Mild barrier disruption; allergen penetration COPD: Cigarette smoke–induced injury Bronchiectasis: Severe barrier compromise; recurrent infections	Protective therapies; infection prevention
Corticosteroid resistance	ROS, HDAC2 nitration/inactivation	Reduced steroid efficacy; persistent inflammation	Asthma: Severe, neutrophilic phenotype COPD: Common; contributes to steroid unresponsiveness Bronchiectasis: Less prominent	Adjust anti-inflammatory therapy; consider non-steroid treatments
Systemic effects	ROS spillover, chronic inflammation	Cardiovascular disease, metabolic dysfunction, osteoporosis	Present in all three, but severity may correlate with systemic inflammation burden	Monitor and manage comorbidities proactively

AP-1, activator protein-1; HDAC2, histone deacetylase 2; MUC, mucin gene; NF-κB, factor kappa-light-chain-enhancer of activated B cells; ROS, reactive oxygen species.

ROS exacerbate airway pathology by promoting mucus hypersecretion through the upregulation of mucin genes (MUC), including MUC5AC and MUC5B, contributing to airway obstruction and impaired mucociliary clearance (Kim et al., 2008). This process is tightly regulated by NF-κB and AP-1, highlighting the central role of oxidative signaling in modulating both inflammatory and structural components of airway remodeling.

Barrier dysfunction represents another critical consequence of oxidative stress. ROS disrupt epithelial tight junction proteins, such as occludin and claudins, resulting in increased airway permeability, heightened susceptibility to infection, and amplification of secondary inflammation across asthma, COPD, and bronchiectasis (Yamamoto et al., 2021).

Oxidative stress also underpins corticosteroid resistance, particularly in severe asthma and COPD, because it induces nitration and functional inactivation of HDAC2, impairing repression of pro-inflammatory genes and diminishing steroid efficacy, thereby perpetuating persistent airway inflammation despite therapy (Barnes, 2009).

Importantly, the impact of oxidative stress extends beyond the lungs, contributing to systemic comorbidities such as cardiovascular disease, metabolic dysfunction, and osteoporosis. Chronic airway inflammation, coupled with ROS spillover into the circulation, drives systemic inflammation, endothelial dysfunction, and dysregulated metabolic signaling, providing a mechanistic link for extra-pulmonary manifestations observed in asthma, COPD, and bronchiectasis (Sinden and Stockley, 2010).

Collectively, these observations underscore the importance of redox imbalance in chronic airway diseases, integrating local airway pathology with systemic consequences, and highlight the potential of antioxidant-based interventions as adjunctive therapeutic strategies.

While many biomarkers are shared across asthma, COPD, and bronchiectasis, disease-specific patterns exist due to differences in predominant inflammatory cells, sources of ROS, and tissue damage. Table 3 summarizes key oxidative stress biomarkers, their sample sources, and their relevance across these three conditions.

**TABLE 3:** Biomarkers of oxidative stress across asthma, COPD, and bronchiectasis.

Biomarker	Type/Function	Sample source	Disease(s)	Clinical/Mechanistic significance
FeNO	Nitrosative stress; surrogate for iNOS activity	Exhaled breath	Asthma	Reflects airway inflammation and oxidative/nitrosative stress; used in asthma monitoring
8-Isoprostane	Lipid peroxidation product	Exhaled breath condensate	Asthma	Correlates with disease severity and poor control; marker of airway oxidative damage
MDA (malondialdehyde)	Lipid peroxidation product	Plasma, exhaled breath condensate, sputum	Asthma, bronchiectasis	Elevated in uncontrolled/severe disease; indicates oxidative stress
GSH/GSSG ratio	Antioxidant status	Plasma, airway samples	Asthma, COPD, bronchiectasis	Low GSH or GSH/GSSG ratio indicates impaired antioxidant defense
ONOO^−^/Nitrotyrosine	Nitrosative stress; protein modification	Sputum, bronchoalveolar lavage	Asthma, bronchiectasis	Elevated levels reflect protein nitration; marker of nitrosative stress
H_2_O_2_	ROS; oxidative stress indicator	Exhaled breath condensate	COPD	Validated marker of airway oxidative stress; elevated during exacerbations
8-OHdG	Oxidative DNA damage	Urine, serum, sputum	COPD, bronchiectasis	Indicates DNA oxidation; elevated in systemic and airway samples
F2-isoprostanes (iPF2α-III)	Lipid peroxidation product	Urine, sputum	COPD, bronchiectasis	Robust index of oxidant stress; correlates with disease burden
4-HNE	Lipid peroxidation product	Sputum, exhaled breath condensate	Bronchiectasis	Local oxidative damage marker; technically difficult to quantify due to rapid metabolism
Advanced oxidation protein products	Protein oxidation	Plasma, sputum	Bronchiectasis	Indicates oxidative protein modification; systemic marker of oxidative stress
Protein carbonyls	Protein oxidation	Plasma, sputum	Bronchiectasis	Reflects protein oxidative damage; systemic marker
Antioxidant enzymes (SOD, GPx, Catalase)	Enzymatic defense against ROS	Plasma, airway samples	Bronchiectasis, COPD, asthma	Reduced activity indicates impaired antioxidant defense
Vitamin C and E	Non-enzymatic antioxidants	Plasma/serum	COPD, bronchiectasis	Reduced levels reflect chronic oxidative stress

GPx, glutathione peroxidase; GSH, glutathione; GSSG, oxidized GSH; H_2_O_2_, hydrogen peroxide; iNOS, inducible nitric oxide synthase; ONOO^−^, peroxynitrite; ROS, reactive oxygen species; SOD, superoxide dismutase; 4-HNE, 4-hydroxynonenal; 8-OHdG, 8-hydroxy-2′-deoxyguanosine.

## COVID-19 and redox signaling in the airways

Even after the pandemic, it remains important to discuss redox signaling in the context of coronavirus disease 2019 (COVID-19), as redox imbalance is a key driver of acute and long-term pulmonary pathology following severe acute respiratory syndrome coronavirus 2 (SARS-CoV-2) infection.

Characterized by a wide spectrum of clinical manifestations ranging from mild upper airway symptoms to viral pneumonia, acute respiratory distress syndrome, and respiratory failure, acute respiratory illness caused by SARS-CoV-2 is known as COVID-19. Standard treatments in the acute phase include supportive care, oxygen supplementation, and, for hypoxemic patients, systemic corticosteroids (particularly dexamethasone at a daily dose of 6 mg) or antiviral therapies (nirmatrelvir/ritonavir for high-risk outpatients and remdesivir for hospitalized patients) (Bhimraj et al., 2024). In selected cases, treatments include immunomodulatory therapies, such as tocilizumab or baricitinib. Since 2020, over 774 million individuals worldwide have been affected by the virus (as of February 2024), with incidence rates closely linked to epidemic waves and viral variants (Gregory and Hall, 2024). However, these figures likely represent significant underestimates due to undiagnosed or unreported cases. A substantial proportion of individuals experience persistent symptoms following an acute infection, a condition referred to as post-COVID condition or long COVID. Respiratory manifestations include chronic dyspnea, cough, reduced exercise tolerance, impaired gas exchange (particularly diffusion capacity), and radiological abnormalities such as ground-glass opacities and fibrotic-like changes (Xie et al., 2025). Management is largely supportive and multidisciplinary, focusing on pulmonary rehabilitation, breathing control techniques, and treatment of residual inflammation or fibrosis when present (Singh et al., 2023). The prevalence of long COVID varies widely depending on the definition and population studied. Among SARS-CoV-2 survivors followed for at least 6 months, the global prevalence of long COVID is 18.0% (Halas et al., 2025). Rates are significantly higher in individuals who were hospitalized during the acute phase of the infection. Prior hospitalization confers an odds ratio of 2.35 for developing persistent symptoms. Recent data from 2024 to 2025 suggest incidence proportions of 4% in children and 10%–26% in adults, with an excess incidence of 1.5% in children and 5%–6% in adults compared to controls (Mandel et al., 2025).

Oxidative stress has been implicated in persistent airway inflammation, fibrosis, and impaired lung function, contributing not only to the severity of acute respiratory distress syndrome but also to the development of pulmonary fibrosis and chronic sequelae, including long COVID (Yang et al., 2024). Consistent with this framework, SARS-CoV-2 infection disrupts cellular redox homeostasis, leading to oxidative and nitrosative stress that exacerbates inflammation, endothelial dysfunction, and tissue injury in the respiratory tract (Cazzola et al., 2021; Alam and Czajkowsky, 2022) (Table 4).

**TABLE 4:** Redox disturbances in COVID-19: mechanisms, consequences, and clinical implications.

Aspect	Mechanisms/Observations	Consequences	Clinical implications
ROS/RNS sources	• Mitochondrial dysfunction • Activated inflammatory cells (e.g., neutrophils) • NADPH oxidases	Elevated ROS/RNS production, oxidative stress	Drives acute lung injury and systemic complications
Suppression of antioxidant defenses	• Impaired Nrf2-driven transcription • Reduced GSH and SOD activity	Loss of redox homeostasis	Increased vulnerability to oxidative damage
Acute pulmonary effects	• Oxidative damage to alveolar epithelium and endothelium • NET-derived oxidants amplify injury	Vascular permeability, pulmonary edema, impaired gas exchange	Acute lung injury, ARDS
Thrombotic effects	• Endothelial dysfunction • NET-associated ROS promote platelet activation	Microvascular thrombosis	Severe disease, multi-organ involvement
Long COVID (redox dysregulation)	• Persistent mitochondrial dysfunction • Elevated serum MDA and hydroperoxides • Ongoing neutrophil activation, NETs • ROS-driven fibrogenic signaling	Chronic oxidative and inflammatory state Airway remodeling	Persistent dyspnea, cough, airway hyperresponsiveness
Systemic sequelae	• Impaired mitochondrial energy metabolism • Dysautonomia linked to oxidative stress	Fatigue, dysautonomia (e.g., POTS)	Prolonged post-viral syndrome, impaired quality of life

ARDS, acute respiratory distress syndrome; GSH, glutathione; MDA, malondialdehyde; NADPH, nicotinamide adenine dinucleotide phosphate; NET, neutrophil extracellular trap; Nrf2, nuclear factor-erythroid 2 p45-related factor 2; POTS, postural orthostatic tachycardia syndrome; RNS, reactive nitrogen; ROS, reactive oxygen species; SOD, superoxide dismutase.

At the mechanistic level, accumulating *in vitro* and *in vivo* evidence demonstrates that SARS-CoV-2 directly impairs key antioxidant defense pathways. In particular, infection downregulates NRF2 protein levels and NRF2-dependent gene expression in human airway epithelial cells and in the lungs of infected mice, independently of proteasomal degradation and interferon signaling (Qu et al., 2023). The functional relevance of this pathway is supported by observations that Nrf2-deficient mice develop more severe disease and heightened lung inflammation, highlighting the protective role of NRF2 during SARS-CoV-2 infection (Qu et al., 2023). Furthermore, the viral ORF6 protein has been shown to directly inhibit NRF2 nuclear translocation and suppress NRF2-regulated antioxidant gene expression, resulting in increased intracellular ROS production (De Angelis et al., 2023).

In parallel, SARS-CoV-2 infection induces profound mitochondrial dysfunction, characterized by inhibition of oxidative phosphorylation, increased mitochondrial ROS generation, and release of mitochondrial DNA, which amplifies innate immune activation and inflammatory signaling (Guarnieri et al., 2024). These effects are mediated, at least in part, by viral proteins such as ORF8 and ORF10, which alter both nuclear and mitochondrial gene expression and promote metabolic reprogramming, potentially contributing to the persistence of symptoms observed in long COVID (Guarnieri et al., 2024). Consistent with these experimental findings, clinical and translational studies have reported increased ROS levels and reduced GSH availability in immune cells and respiratory tissues from COVID-19 patients, linking systemic redox imbalance to disease severity and post-acute sequelae (Tavassolifar et al., 2023).

Collectively, these data provide direct mechanistic evidence that SARS-CoV-2 actively dysregulates the NRF2 antioxidant axis and mitochondrial function, establishing a pro-oxidant airway environment that contributes to acute lung injury and may underlie the persistent inflammation and tissue damage characteristic of long COVID (Cazzola et al., 2021; Alam and Czajkowsky, 2022).

### Consequences for the airways and systemic disease

The clinical consequences of oxidative stress extend beyond acute viral pathology to include airway-specific and systemic manifestations. In the context of acute lung injury and acute respiratory distress syndrome, oxidative damage to the alveolar epithelium and endothelium increases vascular permeability, promotes pulmonary edema, and impairs gas exchange. Observational studies have demonstrated correlations between biomarkers of oxidative stress and disease severity in this setting (Xie et al., 2024). Oxidative stress also contributes to the prothrombotic environment characteristic of severe SARS-CoV-2 infection, as endothelial injury and neutrophil extracellular trap (NET)-derived oxidants amplify platelet activation and drive microvascular thrombosis (Xie et al., 2024).

Of particular significance is the association between dysregulated redox signaling and the post-acute sequelae of SARS-CoV-2 infection. Quantitative studies have demonstrated elevated levels of serum MDA and total hydroperoxides in individuals with long COVID (Stufano et al., 2023). Impaired mitochondrial energy production and increased ROS generation have been observed months after infection (Chen et al., 2023). Nrf2-mediated antioxidant responses are persistently impaired, accompanied by ongoing oxidative and inflammatory signatures (lower GSH and SOD activity), even after viral clearance (Toscanelli et al., 2025; Galli et al., 2022). Residual neutrophilic activation and NETs in the airways, which perpetuate ROS release, mucus abnormalities, and airway epithelial injury, have been observed in patients with persistent pulmonary sequelae (George et al., 2022). In addition, evidence has been presented demonstrating the contribution of ROS-driven fibrogenic signaling to airway remodeling in long COVID cohorts (Yang et al., 2024).

Post-acute sequelae of SARS-CoV-2 infection are characterized by symptoms such as persistent dyspnea and cough. These symptoms are associated with ongoing oxidative stress, mitochondrial dysfunction, and chronic inflammation, which can impair gas exchange and airway function (Sherif et al., 2023). Redox dysregulation contributes to airway hyperresponsiveness and can exacerbate underlying respiratory diseases, such as asthma, COPD, and bronchiectasis, by promoting airway inflammation, epithelial injury, and increased susceptibility to triggers (Checa and Aran, 2020). Systemic fatigue and dysautonomia, including postural orthostatic tachycardia syndrome, are also frequently reported (Gopinathannair et al., 2024). These symptoms are thought to result from mitochondrial dysfunction, persistent immune activation, and autonomic nervous system imbalance driven by oxidative and inflammatory stress (Gopinathannair et al., 2024).

## Therapeutic implications

Understanding the interplay between redox dysregulation, inflammatory signaling, and epigenetic alterations provides opportunities for therapeutic intervention.

### Redox-targeting therapeutics

Redox-targeting therapeutics for chronic airway diseases include several classes of agents that modulate oxidative stress and restore redox homeostasis (Table 5).

**TABLE 5:** Redox-targeting therapeutics in chronic arway diseases.

Therapeutic class	Representative agents	Mechanism of action	Key evidence/Clinical insights
Thiol-based antioxidants	N-acetylcysteine (NAC), erdosteine, carbocysteine, fudosteine	Replenish intracellular GSH, scavenge ROS/RNS, support mitochondrial redox	NAC increases GSH, SOD activity, reduces ROS in alveolar macrophages; erdosteine limits O_2_•^−^, H_2_O_2_, NO, protects against DNA damage; Carbocysteine and fudosteine reduce oxidative markers and airway injury; clinically improves redox balance in COPD
NADPH oxidase inhibitors	General NOX inhibitors	Inhibit NOX enzymatic activity to reduce ROS generation	Preclinical models show decreased ROS-mediated inflammation; limited isoform selectivity may cause off-target effects
Nrf2 activators	Sulforaphane, synthetic electrophilic activators	Promote Nrf2 nuclear translocation, boost GSH/NADPH synthesis, stabilize redox-sensitive transcription factors	Enhances cellular antioxidant capacity and stress adaptation; clinical translation limited by poor solubility, rapid metabolism, and off-target protein interactions
Mitochondria-targeted antioxidants	AntiOxCIN, MitoQ	Accumulate in mitochondria, scavenge mitochondrial ROS, support mitophagy/biogenesis, indirectly activate Nrf2	Reduce mitochondrial oxidative stress in preclinical models; uptake limited in damaged mitochondria, low oral bioavailability
SOD mimetics	AEOL 10150, M40419	Catalytically neutralize superoxide radicals	Preclinical reduction of oxidative injury; clinical translation limited
Spin trapping agents	α-Phenyl-N-tert-butyl nitrone	Stabilize free radicals, interrupt oxidative cascades	Preclinical efficacy; limited clinical data
Natural antioxidants	Resveratrol, curcumin, quercetin, sulforaphane	Scavenge ROS, modulate redox-sensitive pathways	Preclinical anti-inflammatory/antioxidant effects; inconsistent clinical efficacy

COPD, chronic obstructive pulmonary disease; GSH, glutathione; H_2_O_2_, hydrogen peroxide; NADPH, nicotinamide adenine dinucleotide phosphate; NO, nitric oxide; Nrf2, nuclear factor-erythroid 2 p45-related factor 2; NOX, NADPH, oxidase; O_2_•^−^, superoxide anion; ROS, reactive oxygen species; SOD, superoxide dismutase.

The most clinically established agents are thiol-based antioxidants such as N-acetylcysteine (NAC), carbocysteine, erdosteine, and fudosteine. These agents increase endogenous GSH and directly scavenge ROS (Cazzola et al., 2019; Cazzola et al., 2021).

NAC contributes to cellular redox regulation primarily through indirect mechanisms. Although it supplies sulfhydryl group (-SH) groups and provides cysteine for GSH synthesis, NAC’s direct reactivity with major physiological oxidants such as H_2_O_2_ and O_2_•^−^ is too slow to exert meaningful scavenging activity (Ezeriņa et al., 2018). Rather, NAC’s antioxidant effects are more plausibly attributed to replenishing intracellular GSH and potentially generating hydrogen sulfide, which increases in mitochondrial sulfane sulfur. Functionally, NAC enhances antioxidant defenses in experimental COPD models by increasing total antioxidant capacity, GSH levels, and SOD activity while reducing lipopolysaccharide-induced pro-oxidant mediators and markers of oxidative damage (Cazzola et al., 2017). In clinical settings, NAC improves systemic and airway redox balance by elevating GSH concentrations and decreasing ROS production by alveolar macrophages and exhaled H_2_O_2_ (Cazzola et al., 2019).

Erdosteine also demonstrates significant antioxidant activity (Cazzola et al., 2020). It reduces the production of O_2_•^−^, H_2_O_2_, NO, and lysosomal enzymes from activated macrophages. Its active metabolite, MET1, effectively limits intracellular ROS and protects against H_2_O_2_-induced oxidative stress and DNA damage (Hosoe et al., 2002). Across experimental models, erdosteine consistently mitigates oxidative tissue injury. In COPD patients, it improves the oxidant-antioxidant balance, reduces exercise-induced oxidative stress, and lowers ROS and 8-isoprostane levels during acute exacerbations (Dal Negro et al., 2018).

Carbocysteine exhibits weaker direct scavenging capacity due to its thioether-based reducing activity but still produces measurable antioxidant effects (Nogawa et al., 2009), including significant reductions in exhaled 8-isoprostane in COPD patients (Carpagnano et al., 2004).

Fudosteine exerts its antioxidant mechanism primarily by directly scavenging ROS and RNS, including O_2_•^−^ and ONOO^−^, thereby reducing oxidative and nitrative stress in airway tissues (Takahashi et al., 2001; Osoata et al., 2009a). These antioxidant actions have been associated with improved oxidative stress markers and reduced airway injury in preclinical models (He et al., 2022).

NADPH oxidase inhibitors, Nrf2 activators, and mitochondria-targeted antioxidants each modulate cellular redox tone through distinct, well-characterized pharmacodynamic mechanisms. NADPH oxidase inhibitors act by suppressing the enzymatic activity of NOX isoforms, thereby reducing ROS generation and dampening downstream redox signaling (Koju et al., 2019). Nrf2 activators enhance endogenous antioxidant capacity by promoting the Nrf2 nuclear translocation and activation (Kumar and Ramanarayanan, 2025). This increases the synthesis of GSH, NADPH, and other redox buffers, which stabilizes redox-sensitive transcription factors and supports cellular adaptation to oxidative stress. Mitochondria-targeted antioxidants (such as AntiOxCIN) accumulate within mitochondria, where they directly scavenge mitochondrial ROS and support mitochondrial quality control mechanisms such as mitophagy and biogenesis (Amorim et al., 2022). These agents can also indirectly activate Nrf2-dependent antioxidant responses, further enhancing cellular resilience to oxidative injury.

Mitochondrial dysfunction is a critical pathogenic mechanism in chronic airway diseases, particularly COPD and asthma, characterized by reduced mitochondrial membrane potential, increased mitochondrial ROS production, decreased ATP content, and impaired respiratory chain complex expression. In COPD, airway smooth muscle cells and epithelial cells exhibit significant mitochondrial dysfunction that drives inflammation, airway remodeling, corticosteroid resistance, and cellular senescence (Wiegman et al., 2015; Fairley et al., 2023; Li and Liu, 2024). Mitochondria-targeted antioxidants such as MitoQ have demonstrated efficacy in preclinical models by reversing mitochondrial dysfunction, reducing airway inflammation and hyperresponsiveness, and protecting against oxidative damage in both COPD and asthma (Wiegman et al., 2015; Agrawal and Mabalirajan, 2016; Fairley et al., 2023; Huang et al., 2023).

Despite their therapeutic potential, all three classes face significant pharmacokinetic challenges. These include limited tissue penetration, rapid metabolic degradation, and off-target effects due to the widespread distribution of redox enzymes (Chocry and Leloup, 2020; Sezgin Bayindir et al., 2025). Poor solubility, instability, and extensive pre-systemic metabolism often lead to low oral bioavailability and accelerated clearance. This requires higher or repeated dosing, which increases the risk of toxicity.

Additional class-specific limitations further complicate development. NADPH oxidase inhibitors often lack isoform selectivity, which risks the unintended inhibition of other NOX isoforms or unrelated enzymes (Cifuentes-Pagano et al., 2014). Nrf2 activators commonly exhibit low solubility, extensive pre-systemic metabolism, rapid elimination, and poor oral bioavailability (Sezgin Bayindir et al., 2025). This necessitates advanced formulation strategies to overcome these barriers and also address the concern of off-target effects. Many electrophilic activators react with redox-sensitive cysteines in proteins other than Keap1, which further complicates their safety profile.

Mitochondria-targeted antioxidants face substantial translational challenges from preclinical to clinical application. Although *in vitro* studies demonstrate several-hundredfold accumulation of compounds such as MitoQ, MitoTEMPO, and SkQ1 in the mitochondrial matrix compared to cytosol, oral or parenteral dosing *in vivo* yields tissue concentrations typically 10–100 times lower than those used in in vitro experiments, reflecting differences in absorption, distribution, metabolism, excretion, and mitochondrial uptake (Apostolova and Victor, 2015; Fields et al., 2023). Selective targeting of diseased mitochondria is particularly challenging because injured mitochondria often exhibit reduced membrane potential, limiting antioxidant uptake (Oyewole and Birch-Machin, 2015; Jiang et al., 2020). Furthermore, rodents, primates, and humans differ in mitochondrial DNA content, bioenergetic profiles, and drug transporter abundance, all of which affect mitochondrial drug delivery and efficacy (Apostolova and Victor, 2015; Jiang et al., 2020; Fields et al., 2023). Off-target effects of antioxidants are incompletely understood, and high-dose antioxidants can potentially cause unwanted side effects by remodeling ROS signaling; for example, identical doses of MitoQ improved hematopoietic stem cell dysfunction but were toxic to neural stem cells in the same mouse model (Rahman and Rahman, 2018). Clinical translation of mitochondria-targeted antioxidants for chronic airway diseases remains limited, with most human data restricted to early-phase trials or studies in other disease contexts (Jiang et al., 2020; Mason et al., 2022). Additional redox-targeting therapeutics include SOD mimetics (e.g., AEOL 10150, M40419), which catalytically neutralize superoxide radicals, and spin trapping agents such as α-phenyl-N-tert-butyl nitrone, which stabilize free radicals (Barnes, 2020). Both classes of agents offer mechanistic advantages over conventional antioxidants because they target specific reactive species and interrupt key steps in the oxidative stress cascade. However, clinical translation has been limited, and most data come from preclinical models.

Other agents with redox-modulating properties include natural antioxidants (e.g., resveratrol, curcumin, quercetin, and sulforaphane) (Chang et al., 2025). However, dietary antioxidants have not shown consistent clinical efficacy in COPD (Barnes, 2020).

In any case, conventional antioxidants lack specificity for mitochondrial compartments, often leading to subtherapeutic concentrations at target sites and potential systemic side effects (Plotnikov and Zorov, 2019). Although general antioxidants can modulate systemic redox balance, their non-specific distribution, context-dependent effects, and potential to disrupt physiological ROS signaling limit clinical utility (Alum et al., 2025). In some cases, antioxidants may even exacerbate disease, promote cellular dysfunction, or contribute to toxicity by shifting the redox balance in unintended directions (Alum et al., 2025). This evidence underscores the need for targeted antioxidant strategies that selectively modulate pathological oxidative stress while preserving normal redox-dependent cellular functions.

Recent advances have expanded redox-targeted therapies beyond classical drugs to include innovative strategies leveraging nanotechnology and gene-editing tools. Nanodelivery systems, including liposomes, polymeric nanoparticles, dendrimers, and metallic nanoparticles, enable targeted delivery of antioxidants and redox-modulating agents, improving bioavailability, stability, and site-specific action in oxidative stress-driven diseases such as COPD and asthma (Karati et al., 2025). These platforms can be engineered to respond to elevated ROS or GSH levels characteristic of pathological microenvironments, thereby enhancing therapeutic efficacy and minimizing off-target toxicity (Chen M. et al., 2021; Feng et al., 2025; Abbasi et al., 2026). Polyethylene glycol (PEG)-ylated nanoparticles have demonstrated the ability to cross airway mucus barriers and avoid phagocytic uptake by alveolar macrophages, enabling widespread transgene expression in lung parenchymal cells (Chen D. et al., 2021). Nebulized lipid nanoparticles based on degradable ionizable glycerolipids have shown potent pulmonary mRNA delivery with efficient transfection of epithelial cells and therapeutic efficacy in elastase-induced emphysema models (Huang et al., 2025).

In parallel, gene editing technologies, particularly CRISPR/Cas9, are emerging as promising tools for treating chronic respiratory diseases, including COPD and asthma, by correcting genetic mutations, modulating aberrant gene expression, and suppressing pathogenic signaling pathways (Ajaykumar et al., 2025). The CRISPR/Cas9 system has been applied in preclinical models to investigate mechanisms of respiratory diseases and identify novel therapeutic targets, with potential applications in correcting deleterious mutations in patient-derived cells and altering disease-related genes (Moses and Kaur, 2019; Ajaykumar et al., 2025). Inhalation delivery of CRISPR/Cas9 therapeutics offers the advantages of high local drug concentration and minimized systemic exposure; however, efficient delivery remains challenging due to biological barriers, including mucus clearance, enzymatic degradation, and cellular membrane penetration (Chow et al., 2021). Non-viral nanocarriers, including biodegradable poly (beta-amino ester) nanoparticles and PEGylated composite nanoparticles, have demonstrated efficient systemic and local delivery of mRNA and gene-editing machinery to lung epithelial and endothelial cells with high transfection efficiency and minimal toxicity in preclinical models (Kavanagh et al., 2025). However, clinical translation of gene editing for chronic airway diseases remains at an early stage, with ongoing challenges in delivery efficiency, targeting specificity, safety, and long-term efficacy requiring further investigation.

Overall, these approaches represent a rapidly evolving frontier in redox-targeted therapy for chronic airway diseases, addressing the limitations of conventional antioxidants and expanding therapeutic possibilities for oxidative stress-related respiratory disorders through targeted mitochondrial interventions, advanced nanodelivery platforms, and precision gene editing strategies.

### Biomarker-guided therapeutic selection

Recent progress in biomarker discovery has facilitated the incorporation of redox and inflammatory biology into clinical decision-making, particularly for diseases in which oxidative stress and inflammation are key drivers of pathogenesis (Menzel et al., 2021). Biomarkers such as 8-isoprostane, nitrotyrosine, reduced GSH/GSSG ratios, and inflammatory cytokine signatures are increasingly recognized for their capacity to indicate disease activity and categorize patients for targeted therapies (Chamitava et al., 2020). Serial and dynamic quantification of these biomarkers provides real-time insight into pharmacodynamic responses, thereby supporting data-driven dose adjustments and enhancing therapeutic efficacy while limiting adverse effects (Margaritelis, 2023). This approach is particularly relevant in the context of redox-modulating and anti-inflammatory therapies, where individual variability in redox homeostasis may substantially influence clinical outcomes. However, the translational value of these biomarkers depends on rigorous analytical and clinical validation, as well as the availability of cost-efficient, scalable, and user-friendly assay platforms. Overall, biomarker-guided strategies are a significant step toward personalized pharmacotherapy in redox-driven diseases. These strategies enable clinicians to tailor interventions to patients’ individual biological profiles and dynamic treatment responses.

### Therapeutic restoration of redox-mediated HDAC2 dysfunction

A critical therapeutic focus is mitigating the reduction in HDAC2 activity caused by oxidative stress, which results in corticosteroid resistance (Osoata et al., 2009b). Among pharmacologic interventions, statins, low-dose theophylline, and curcumin are the most scientifically supported agents for restoring HDAC2 activity. The effect of statin is mediated through modulation of the mevalonate pathway, which counteracts the reduction in HDAC2 expression and activity induced by oxidative stressors such as hydrogen peroxide (Matera et al., 2018). Simvastatin, in particular, has demonstrated the ability to restore both HDAC2 expression and activity under these conditions, whereas corticosteroids alone fail to reverse such impairment. Low-dose theophylline, administered at sub-bronchodilator concentrations, acts as a direct HDAC2 activator (Cosio et al., 2004). It increases HDAC2 activity in epithelial cells and alveolar macrophages. This reverses corticosteroid resistance in conditions such as COPD and asthma, which are characterized by increased oxidative stress. Curcumin exerts its effects post-translationally by inhibiting HDAC2 protein degradation (Meja et al., 2008). Consequently, it preserves both enzymatic activity and expression and effectively reverses steroid insensitivity induced by oxidative stress.

## Future directions to improve the understanding of redox signaling in chronic airway diseases

Improving our understanding of redox signaling in chronic airway diseases is crucial because oxidant-antioxidant balance is not just a marker of damage but an active regulator of disease biology (Table 6).

**TABLE 6:** Future directions to improve the understanding of redox signaling in chronic airway diseases.

Focus area	Key future directions	Purpose/expected impact
Specific redox modifications	Shift from bulk ROS measurement to identifying specific redox modifications (e.g., cysteine sulfenylation, S-glutathionylation, S-nitrosylation) using redox-proteomics and activity-based probes.	Provides mechanistic insight into redox-regulated proteins and signaling pathways, beyond nonspecific oxidative stress markers.
Single-cell and spatial analysis	Apply single-cell transcriptomics, single-cell redox-proteomics, and spatial imaging (e.g., redox-sensitive reporters, proximity labeling) to define cell-type-specific redox signatures in epithelium, fibroblasts, macrophages, *etc.*	Reveals intercellular redox communication and heterogeneity within airway tissues.
Advanced human models	Expand use of patient-derived airway organoids, primary epithelial ALI cultures, and precision-cut lung slices under physiological conditions (e.g., pollutants, viral infection).	Improves translation of findings from animal models to human disease contexts.
Real-time redox imaging	Use genetically encoded reporters in conditional mouse models and human *ex vivo* tissues for real-time visualization of H_2_O_2_ and thiol dynamics.	Links dynamic redox signaling events to physiological and therapeutic responses.
Enzymatic sources and pathways	Focus on NADPH oxidase, mitochondrial ROS, NOS/iNOS, Trx/Grx systems, and Nrf2/Keap1 axis using isoform-selective inhibitors or knockouts.	Identifies disease-specific enzymatic drivers and avoids nonspecific antioxidant failures.
Immunity and infection	Study how altered redox signaling affects innate/adaptive immune responses (e.g., macrophage polarization, NETosis) and infection susceptibility using redox-infection models.	Clarifies mechanisms driving exacerbations in asthma and COPD.
Multi-omics integration	Integrate redox-proteomics, phosphoproteomics, metabolomics, and single-cell RNA data with causal modeling to identify network hubs and druggable nodes.	Builds systems-level understanding and identifies robust therapeutic targets.
Biomarker development	Develop and validate redox biomarkers (exhaled NO, BAL/sputum GSH/GSSG, plasma redox proteome, sulfenylation assays) for patient stratification.	Enables precision medicine by identifying redox endotypes and monitoring target engagement.
Targeted redox modulators	Replace generic antioxidants with pathway-specific modulators (Nrf2 activators, Trx/Grx modulators, NOX inhibitors, mitochondrial ROS modulators, S-nitrosothiol donors).	Restores physiological redox signaling without disrupting essential functions.
Exposome, aging, and sex differences	Investigate how environmental exposures, smoking, diet, aging, and sex interact with redox pathways through longitudinal cohort studies.	Explains inter-patient variability and guides personalized therapeutic strategies.

ALI, alveolar-liquid interface; BAL, bronchoalveolar lavage; Grx, glutaredoxin; GSH, glutathione; GSSG, oxidized GSH; H_2_O_2_, hydrogen peroxide; iNOS, inducible nitric oxide synthase; NADPH, nicotinamide adenine dinucleotide phosphate; NET, neutrophil extracellular trap; NOS, nitric oxide synthase; Nrf2, nuclear factor-erythroid 2 p45-related factor 2; ROS, reactive oxygen species; SNO, S-nitrosothiol; Trx, thioredoxin.

Firstly, it is crucial to transition from focusing on bulk ROS measurements to analyzing redox modifications (e.g., cysteine sulfenylation, S-glutathionylation, S-nitrosylation) on proteins that undergo changes in disease states and the subsequent impact on signaling. The use of targeted redox-proteomics and activity-based probes is recommended as an alternative to relying exclusively on total ROS/GSH assays (Mumby and Adcock, 2022).

The application of single-cell transcriptomics and single-cell redox proteomics, in conjunction with spatial imaging techniques such as redox-sensitive fluorescent reporters and proximity labeling, is essential for the resolution of cell-type-specific redox signatures (Thannickal, 2020). These signatures, which include those from various cell types such as epithelium, fibroblasts, macrophages, neutrophils, and innate lymphoid cells, are crucial for understanding the complex dynamics of redox signaling within biological systems. A concomitant analysis of lung slice and organoid spatial readouts is necessary to ensure the preservation of the microenvironment (Thannickal, 2020). This phenomenon elucidates the mechanism by which neighboring cells exchange redox signals, such as H_2_O_2_ and S-nitrosothiol (SNO).

The utilization of patient-derived airway organoids, primary human airway epithelial alveolar-liquid interface cultures, and precision-cut lung slices should be expanded in order to facilitate the study of redox signaling under physiologic oxygen gradients and realistic exposures, such as cigarette smoke, pollutants, and viral infection (Thannickal, 2020).

The utilization of genetically encoded redox reporters, including reduction-oxidation sensitive green fluorescent protein variants and the HyPer family of reporters, which are established tools for real-time visualization of H_2_O_2_ and thiol redox dynamics in living systems, in conditional mouse models and human *ex vivo* tissue facilitates the acquisition of high-resolution, real-time data regarding H_2_O_2_ and thiol redox dynamics (Li et al., 2025). The integration of these data with functional physiological measurements provides a mechanistic understanding of redox-dependent processes during disease and therapy.

The prioritization of studying specific enzymatic sources of ROS, such as NADPH oxidase isoforms (NOX1-5, DUOX1/2), mitochondrial ROS, NOS/iNOS, Trx/glutaredoxin (Grx) systems, and the Nrf2/KEAP1 axis in defined cell types using conditional knockouts or isoform-selective inhibitors is strongly supported by current translational and preclinical evidence (Sies and Jones, 2020; Dao, et al., 2020). This approach is favored over non-specific antioxidant strategies, which have repeatedly failed in clinical trials due to their inability to distinguish between physiological and pathological ROS signaling, sometimes even causing harm by disrupting essential redox-dependent cellular functions.

A comprehensive examination of the impact of altered airway redox signaling on innate and adaptive immunity (macrophage polarization, neutrophil NETosis, and antigen-presenting cell function) and host defense against bacteria and viruses is a need for a comprehensive understanding of the mechanisms underlying COPD and asthma exacerbations (Braunstein et al., 2025). The utilization of combined infection and redox perturbation models is a priority.

The implementation of integrated datasets, encompassing redox-proteomics, phosphoproteomics, metabolomics, and single-cell RNA, in conjunction with causal/inference modeling, is essential for the identification of redox-regulated network hubs and druggable nodes (Mumby and Adcock, 2022). It is vital to prioritize reproducible signatures across cohorts.

The development and validation of redox biomarkers for patient selection and target engagement is essential for the advancement of this field, not least in the selection of appropriate patients for clinical trials (Meng et al., 2021). Exhaled NO species (FeNO and other SNO metrics), bronchoalveolar lavage/induced sputum GSH/GSSG, plasma redox proteome signatures, and targeted PTM (e.g., protein sulfenylation) assays can be used to assess these processes (Margaritelis et al., 2016). The utilization of biomarkers is instrumental in delineating redox endotypes, such as the Nrf2-deficient endotype and the mitochondrial dysfunction endotype, thereby facilitating precision therapy (Morgenstern et al., 2024).

The prevailing notion of generic antioxidants must be overcome. The current consensus in redox medicine is that targeted modulation of physiological redox signaling, rather than non-specific antioxidant therapy, is the preferred strategy for restoring redox homeostasis in disease contexts (Sies and Jones, 2020). This approach includes the use of small molecules and biological products that specifically modulate key redox pathways, such as Nrf2 activators (Wang et al., 2024), Trx/Grx pathway modulators (Zhang et al., 2021), isoform-selective NOX inhibitors (Dao, et al., 2020), mitochondrial-targeted ROS modulators (Oyewole and Birch-Machin, 2015), and SNO donors, when appropriate (Kolesnik et al., 2014). The target involvement of these molecules must be rigorously tested using the biomarkers described above before initiating wider clinical trials.

A comprehensive examination is necessary to elucidate the intricate interplay among lifetime exposures (e.g., pollutants, smoking, diet), biological sex, and aging in relation to airway redox signaling (Agustí et al., 2022). This multifaceted relationship contributes to the emergence of heterogeneous disease phenotypes and treatment responses (Emeny et al., 2021). It is also necessary to design longitudinal cohorts that integrate exposome measures (Vineis et al., 2020). It is anticipated that a better understanding of redox signaling in chronic airway diseases will ultimately lead to improved therapies for these diseases that currently faces with a high unmet need.

## Conclusion

The evidence presented in this article suggests that precision redox medicine may become a superior alternative to traditional, non-specific antioxidant strategies. This transition represents a fundamental paradigm shift from the broad and often ineffective use of general antioxidants to the specific modulation of redox processes. This evolution holds great promise for improving therapeutic outcomes and minimizing unintended effects. However, it is important to note that this is a narrative review, reflecting the authors’ perspectives rather than a comprehensive synthesis of all available evidence. Future empirical and translational research is essential to substantiating these concepts and defining the precise clinical value of targeted redox modulation.
